# Investing in human resources for health: beyond health outcomes

**DOI:** 10.1186/s12960-016-0147-2

**Published:** 2016-08-15

**Authors:** Giorgio Cometto, James Campbell

**Affiliations:** Health Workforce, World Health Organization, Avenue Appia 20, CH-1211 Geneva 27, Switzerland

Human resources for health are necessary to the delivery of health services; only by securing a sufficient, equitably distributed, adequately supported and well-performing health workforce can any health goals and targets set by national governments or the international community be met [1]. In spite of the recognition of this central role in attaining health outcomes, investments in human resources for health have been and still are constrained by the perception that the health economy (and the health workers within it) is a consumptive sector, whose costs governments should strive to contain, rather than a contributor to socio-economic development in its own right. This thematic series sought to examine and broaden the evidence on the contribution of investment in human resources for health to broader development outcomes in other sectors, including synergies with education, finance, employment, gender empowerment and peace building.

The WHO *Global Strategy on Human Resources for Health: Workforce 2030*, adopted at the World Health Assembly in May 2016, articulates one of its objectives around the linkage between investments in the health workforce and “improvements in health outcomes, social welfare, employment creation and economic growth”, arguing that the investment in human resources for health can deliver a triple return of improved health outcomes, global health security and economic growth [2].

And, indeed, evidence from this thematic series underscores the bi-directional nature of the relationship between health workforce investments and the broader socio-economic development features of countries. On the one hand, the capacity of countries to produce health workers is influenced and determined by socio-economic factors, such as income levels, education attainments, emigration rates and availability of health infrastructure, as Squires et al. point out [3]. On the other hand, Scheil-Adlung et al. show that investment in the health workforce can have a major positive impact on socio-economic development, particularly in the world’s poorest countries, where it can increase equity, reduce poverty due to ill health and ultimately contribute to sustainable development and social justice [4].

These findings reinforce recent related analyses showing how health sector employment significantly contributes to productivity growth in other sectors [5] and that it is more resilient than other types of employment during financial downturns [6].

Pálsdóttir et al. examine transformational education approaches, finding that strategies such as training health workers within communities, better aligning skills and competencies with population and health system needs, ensuring a gender-balanced workforce and enhancing inter-professional learning can maximize the social and economic return on investment [7].

Finally, at a time when political instability and violent conflict affect a growing number of people around the world, a new lens to the health workforce discourse entails examining its potential to contribute to wider state-building efforts. According to Witter and co-authors, the development of health cadres and the reintegration of factional health staff post-conflict can be plausibly linked to a strengthened public administration, state-building features and reconciliation efforts [8].

But at the same time, it is critical that HRH investments be tailored to the national setting and its fiscal realities; in this context, it is important to understand the implications of growth in health sector employment on public sector health spending, and reflect this in increasing the fiscal space in line with population needs related to the universal health coverage objectives, as the case study from Serbia by Santric-Milicevic et al. illustrates [9].

The impact of investment can be maximized by improving the efficiency of HRH spending: more comprehensive and reliable data and evidence are required to rationalize health workforce planning and management, as evidenced by complementary case studies from Uganda. At the facility level, Namaganda and colleagues show how the adoption of workload-based norms can contribute to redistributing existing health workers, improving staffing distribution and, consequently, equity in access to health services [10]. At the health system level, Driessen and co-authors find that improved health workforce evidence can allow the health sector to recruit the best candidates, train employees in needed skills and deploy personnel based on actual demand patterns [11].

Overall, the case for investing in human resources for health has never been stronger, with the potential for a positive impact going far beyond the health sector; and examples exist of how better evidence and the use of new tools and approaches can maximize the returns on this investment. While these elements together provide the technical foundations for greater and smarter health workforce investments, these will only materialize with the high-level political commitment of governments. The recently launched United Nations High Level Commission on Health Employment and Economic Growth provides an unprecedented opportunity to leverage political commitment, exploring and formulating recommendations on how investments in HRH represent a critical strategy to redress existing and projected inequities in access to health workers, while at the same time stimulating the creation of jobs in the health and social sectors for inclusive economic development [12].

## References

[1] Campbell J, Dussault G, Buchan J, Pozo-Martin F, Guerra Arias M, Leone C (2013). A universal truth: no health without a workforce. Forum report, third Global Forum on Human Resources for Health, Recife, Brazil.

[2] WHO (2016). Global Strategy on Human Resources for Health: Workforce 2030.

[3] Squires A, Uyei J, Beltrán-Sánchez H, Jones S. Examining the influence of country level and health system factors on nursing and physician personnel production. Hum Resour Health. 2016. doi: 10.1186/s12960-016-0145-4. 10.1186/s12960-016-0145-4 PMC4983794 27523185

[4] Scheil-Adlung X, Thorsten Behrendt T, Wong L (2015). Health sector employment: a tracer indicator for universal health coverage in national social protection floors. Hum Resour Health.

[5] Arcand, JL, Araujo EC, Menkulasic G, Weber M. Health sector employment, health care expenditure and economic growth: what are the associations? Washington DC: World Bank. (in press).

[6] Employment polarisation and job quality in the crisis: European Jobs Monitor 2013. Dublin: Eurofound; 2013. www.eurofound.europa.eu/pubdocs/2013/04/en/1/EF1304EN.pdf. Accessed 9 June 2016.

[7] Pálsdóttir B, Barry J, Bruno A, Barr H, Clitero A, Cobb N et al Training for impact: the socio-economic impact of a fit for purpose health workforce on communities. Hum Resour Health. 2016. doi: 10.1186/s12960-016-0143-6. 10.1186/s12960-016-0143-6 PMC4983779 27523088

[8] Witter S, Falisse JB, Bertone MP, Alonso-Garbayo A, Martins J, Salehi A (2015). State-building and human resources for health in fragile and conflict-affected states: exploring the linkages. Hum Resour Health.

[9] Santric-Milicevic M, Vasic V, Terzic-Supic Z. Do health care workforce, population and service provision significantly contribute to the total health expenditure? An econometric analysis of Serbia. Hum Resour Health. 2016. doi: 10.1186/s12960-016-0146-3. 10.1186/s12960-016-0146-3 PMC4986341 27526854

[10] Namaganda G, Oketcho V, Maniple E, Viadro C (2015). Making the transition to workload-based staffing: using the workload indicators of staffing need method in Uganda. Hum Resour Health.

[11] Driessen J, Settle D, Potenziani D, Tulenko K, Kabocho T, Wadembere I (2015). Understanding and valuing the broader health system benefits of Uganda’s national Human Resources for Health Information System investment. Hum Resour Health.

[12] United Nations (2016). High Level Commission on Health Employment and Economic Growth.

